# Adaptive Complex Variational Mode Decomposition for Micro-Motion Signal Processing Applications

**DOI:** 10.3390/s21051637

**Published:** 2021-02-26

**Authors:** Saiqiang Xia, Jun Yang, Wanyong Cai, Chaowei Zhang, Liangfa Hua, Zibo Zhou

**Affiliations:** Air Force Early Warning Academy, Wuhan 430019, China; yangjem@126.com (J.Y.); caiwanyong2021@163.com (W.C.); zhangchaowei2019@163.com (C.Z.); lfhua2002@163.com (L.H.); zibo_travel@163.com (Z.Z.)

**Keywords:** narrow-band radar, micro-motion, signal separation, optimal decomposition layer, complex variational mode decomposition, signal reconstruction

## Abstract

In order to suppress the strong clutter component and separate the effective fretting component from narrow-band radar echo, a method based on complex variational mode decomposition (CVMD) is proposed in this paper. The CVMD is extended from variational mode decomposition (VMD), which is a recently introduced technique for adaptive signal decomposition, limited to only dealing with the real signal. Thus, the VMD is extended from the real domain to the complex domain firstly. Then, the optimal effective order of singular value is obtained by singular value decomposition (SVD) to solve the problem of under-decomposition or over-decomposition caused by unreasonable choice of decomposition layer, it is more accurate than detrended fluctuation analysis (DFA) and empirical mode decomposition (EMD). Finally, the strongly correlated modes and weakly correlated modes are judged by calculating the Mahalanobis distance between the band-limited intrinsic mode functions (BLIMFs) and the original signal, which is more robust than the correlation judgment methods such as computing cross-correlation, Euclidean distance, Bhattachryya distance and Hausdorff distance. After the weak correlation modes are eliminated, the signal is reconstructed locally, and the separation of the micro-motion signal is realized. The experimental results show that the proposed method can filter out the strong clutter component and the fuselage component from radar echo more effectively than the local mean decomposition (LMD), empirical mode decomposition and moving target indicator (MTI) filter.

## 1. Introduction

Micro-motion [1,2,3,4] refers to the vibration, rolling and rotation of the target in addition to rigid motion. Since the concept of micro-motion was proposed by professor V.C. Chen of the US military laboratory, the micro-motion characteristics in narrow-band radar echo have been widely studied and it provides a new solution for radar target detection and recognition. In practical application, radar echo is usually mixed with noise and clutter, and the traditional clutter suppression method of moving target indicator (MTI) combined with coherent accumulation usually causes excessive attenuation of doppler components of target near-zero frequency, which leads to the degradation of radar detection performance [5], and it is difficult to obtain effective target information. Therefore, it is of great significance to use effective echo separation method to suppress clutter and fuselage components, extracting fretting component of a target for improving the recognition ability of narrow-band radar.

Radar echo is a case in non-linear and non-stationary signal. In order to separate these signals, subspace-based clutter suppression and adaptive time-frequency analysis are widely used. The typical methods of subspace-based clutter suppression are singular-value decomposition (SVD) [6], principal component analysis (PCA) [7] and independent component analysis (ICA) [8]. Reference [9] comprehensively compares the application of the above-mentioned three methods of clutter suppression and validates the effectiveness of these methods through the measured data. However, most subspace-based clutter suppression methods still have several typical problems: (1) Supposing the environment model, once the target’s environment and assumptions do not match, the clutter suppression performance will be less effective. (2) It is assumed that the signal subspace and the clutter subspace are independent, but in fact, the two subspaces are usually multi-dimensional and have some correlation [10,11]. (3) The clutter subspace is constructed by assuming the eigenvectors corresponding to the largest eigenvalues of echo’s co-variance matrix, which are usually selected by manual or simple threshold setting. The typical adaptive time-frequency analysis methods are empirical mode decomposition (EMD) [12,13,14] and local mean decomposition (LMD) [15,16,17]. Taking advantage of the time-scale characteristics of the signal, these methods do not need to set the basis function to decompose the signal in advance, and these methods have unique advantages in stabilizing the unstable signal. However, due to the use of recursive decomposition, the estimation error of decomposition will be continuously transmitted, that results in modal aliasing and end-point effect. It is difficult to decompose the adjacent frequency components. Moreover, these methods need large amounts of calculation, and the reconstruction method can only be used for a few special occasions. In order to solve these problems, Reference [18] proposed the variational mode decomposition (VMD) method, which is a non-recursive adaptive time-frequency analysis method. The method assumes that each mode is tightly clustered around a central frequency, then the solving of mode bandwidth is transformed into a constrained optimization processing. Not only can the mode aliasing and the end-point effect be solved well, but also the calculation amount is small. But this method also has some defects: (1) Only real signals can be processed, (2) the number of decomposition layers needs to be set in advance, and unreasonable layers will lead to over-decomposition or under-decomposition situations, and (3) mode selection is difficult in signal reconstruction. 

Aiming at the deficiency of VMD in processing narrow-band radar echo, this paper proposes an adaptive complex variational mode decomposition method, which combines VMD with SVD and Mahalanobis distance (MD). The method extends VMD from the real domain to the complex domain to adapt for radar echo processing firstly. Then, the complex signal is used to construct Hank matrix, and SVD is implemented to obtain the optimal decomposition layer of the CVMD method. After that, the correlation is judged by calculating the MD between band-limited intrinsic mode functions (BLIMFs) and the original signal to distinguish the strong correlation mode and the weak correlation mode. Finally, the local reconstruction of the strong correlation mode is implemented to separate the micro-motion signal from clutter and fuselage signal.

## 2. Brief Description of Variational Mode Decomposition

VMD is a solving process of variational problem based on Wiener filter, Hilbert transform and mixed frequency. By iteratively searching the optimal solution of the constrained variational model, a real signal is adaptively transformed into *K* band-limited intrinsic mode functions with special sparsity and independence. Then, the signal is separated to different frequency components, namely the low-frequency components and the high-frequency components are separated effectively. On the basis of the hypothesis that each mode surrounds a central frequency in the frequency domain and the frequency bandwidth is compact, therefore, the key to the implementation of the VMD method is to calculate the center frequency and bandwidth of each mode, and the center frequency is affected by the number of decomposition layer *K*. The steps for solving the constrained variational problem [18] are as follows:

(1) Firstly, the original signal is transformed by Hilbert transform to obtain the analytic signal of each mode component, and the single side spectrum is obtained.

(1) $$\xi_{k}^{’}(t)=(\delta (t)+\frac{j}{\pi t})*u_{k}(t)$$

where δ(⋅) is the Dirac distribution, t is the time script, j is the imaginary unit, $u_{k}(t)=\{u_{1}(t),u_{2}(t),\cdot \cdot \cdot ,u_{K}(t)\}$ is the decomposed BLIMF component and ∗ denotes convolution.

(2) The center frequency $\omega_{k}$ of each modal component $u_{k}(t)$ is estimated and the spectrum is modulated to the corresponding baseband.

(2) $$\xi_{k}(t)=[(\delta (t)+\frac{j}{\pi t})*u_{k}(t)]e^{-j\omega_{k}t}$$

where $\omega_{k}=\{\omega_{1},\omega_{2},\cdot \cdot \cdot ,\omega_{K}\}$ is the center frequency of each BLIMF component.

(3) The square of the l2 norm of the gradient of the modulated signal and the bandwidth of each mode component is estimated. The constrained variational mode is constructed, which is described by the following equation:

(3) $$\underset{\left\{u_{k}\},\{\omega_{k}\right\}}{\min}\{{\sum_{k=1}^{K}\left\|\partial_{t}[(\delta (t)+\frac{j}{\pi t})*u_{k}(t)]e^{-j\omega_{k}t}\right\|}_{2}^{2}\}s.t.\;\sum_{k=1}^{K}u_{k}(t)=x(t)$$

where $\partial_{t}$ represents partial derivation.

(4) By introducing the balancing parameter of the data-fidelity constraint α with great convergence property and the Lagrange multiplier λ(t) with strict constraint performance, the constrained optimization problem is transformed into an unconstrained optimization problem, and the augmented Lagrange expression can be written as follows:

(4) $$\begin{array}{l} L(\{u_{k}(t)\},\{\omega_{k}(t)\},\lambda (t))=\alpha \sum_{k}{\left\|\partial_{t}(\xi_{k}(t))\right\|}_{2}^{2}+{\left\|x(t)-\sum_{k}u_{k}(t)\right\|}_{2}^{2}+\langle \lambda (t),x(t)-\sum_{k}u_{k}(t)\rangle \\ \leq \alpha \sum_{k}{\left\|\partial_{t}(\xi_{k}(t))\right\|}_{2}^{2}+{\left\|x(t)-\sum_{k}u_{k}(t)+\frac{\lambda (t)}{2}\right\|}_{2}^{2}-{\left(\frac{\lambda (t)}{2}\right)}^{2} \end{array}$$

In order to solve the minimum value problem in Equation (4), the $u_{k}^{n+1}(t)$,$\omega_{k}^{n+1}(t)$ and $\lambda^{n+1}(t)$ are alternately updated by the alternative direction method of multipliers (ADMM) to find the saddle point of Equation (4). The solution of constrained variational problem is completed, and the updated formula and convergence conditions of the three variables are shown as follows:

(5) $$\left\{\begin{array}{l} u_{k}^{n+1}(t)\leftarrow \underset{u_{k}(t)}{\arg\min}L(\{u_{i<k}^{n+1}(t)\},\{u_{i\geq k}^{n}(t)\},\{\omega_{i}^{n}(t)\},\lambda^{n}(t))\\ \omega_{k}^{n+1}(t)\leftarrow \underset{\omega_{k}(t)}{\arg\min}L(\{u_{i}^{n+1}(t)\},\{\omega_{i<k}^{n+1}(t)\},\{\omega_{i\geq k}^{n}(t)\},\lambda^{n}(t))\\ \lambda^{n+1}(t)\leftarrow \lambda^{n}(t)+\tau (x(t)-\sum_{k}u_{k}^{n+1}(t))\\ \sum_{k}{\left\|u_{k}^{n+1}(t)-u_{k}^{n}(t)\right\|}_{2}^{2}/{\left\|u_{k}^{n}(t)\right\|}_{2}^{2}<\varepsilon \end{array}\right.$$

where n represents iteration times, i represents the number of modes and $i\in \left[1,K\right]$.

(5) The iterative update of $u_{k}(t)$ and $\omega_{k}(t)$ in the frequency domain.

In view of the difficulty of updating $u_{k}(t)$ and $\omega_{k}(t)$ in the time domain, thus, the update can be realized in the frequency domain, and then transformed to the time domain by inverse Fourier transform. Since $\left(\lambda (t\right)/2{)}^{2}$ does not affect the optimization of $u_{k}(t)$, the optimization problem in Equations (4) and (5) can be transformed into the optimization problem shown in Equation (6), as $u_{k}^{n+1}(t)$, for example:

(6) $$u_{k}^{n+1}(t)=\underset{\left\{u_{k}(t)\right\}}{\arg\min}\{\alpha \sum_{k}{\left\|\partial_{t}(\xi_{k}(t))\right\|}_{2}^{2}+{\left\|x(t)-\sum_{i}u_{i}(t)+\frac{\lambda (t)}{2}\right\|}_{2}^{2}\}$$

For simplicity, the superscripts $\cdot^{n}$ and $\cdot^{n+1}$ on the right-hand side of the equation are ignored. After the Fourier transform, the frequency domain expressions of x(t), $\xi_{k}(t)$, $u_{k}(t)$ and λ(t) are $\hat{x}(\omega )$, ${\hat{\xi }}_{k}(\omega )$, ${\hat{u}}_{k}(\omega )$ and $\hat{\lambda }(\omega )$. From Equations (1) and (2), we can obtain:

(7) $${\hat{\xi }}_{k}(\omega )=[1+\mathrm{sgn}(\omega +\omega_{k})]\cdot u_{k}(\omega +\omega_{k})$$

By using the Parseval or Plancherel Fourier equidistant transform, Equation (6) is transformed to the frequency domain. Here, only the calculation of single value *k* is discussed. The frequency domain expression after removing the symbol $\sum_{k}\cdot$ is written as:

(8) $${\hat{u}}_{{}_{k}}^{n+1}(\omega )=\underset{{\hat{u}}_{k}(\omega )}{\arg\min}\{\alpha {\left\|j\omega [1+\mathrm{sgn}(\omega +\omega_{k})]{\hat{u}}_{k}(\omega +\omega_{k})\right\|}_{2}^{2}+{\left\|\hat{x}(\omega )-\sum_{i}{\hat{u}}_{i}(\omega )+\frac{\hat{\lambda }(\omega )}{2}\right\|}_{2}^{2}\}$$

Let $\omega =\omega +\omega_{k}$, the l2 norm of Equation (8) can be obtained:

(9) $$\begin{array}{l} {\hat{u}}_{{}_{k}}^{n+1}(\omega )=\underset{{\hat{u}}_{k}(\omega )}{\arg\min}\{\int_{-\infty }^{\infty }\left\{\alpha {\left|j(\omega -\omega_{k})[1+\mathrm{sgn}(\omega )]{\hat{u}}_{k}(\omega )\right|}^{2}+{\left|\hat{x}(\omega )-\sum_{i}{\hat{u}}_{i}(\omega )+\frac{\hat{\lambda }(\omega )}{2}\right|}^{2}\right\}d\omega \}\\ =\underset{{\hat{u}}_{k}(\omega )}{\arg\min}\{\int_{0}^{\infty }\left[4\alpha {\left(\omega -\omega_{k}\right)}^{2}{\left|{\hat{u}}_{k}(\omega )\right|}^{2}+2{\left|\hat{x}(\omega )-\sum_{i}{\hat{u}}_{i}(\omega )+\frac{\hat{\lambda }(\omega )}{2}\right|}^{2}\right]d\omega \} \end{array}$$

It can be seen from the above equation that the integrand f(ω) is a positive function, accordingly, the solution of ${\hat{u}}_{{}_{k}}^{n+1}(\omega )=\underset{{\hat{u}}_{k}(\omega )}{\arg\min}\int_{0}^{\infty }f(\omega )d\omega$ can be equivalent to the solution of ${\hat{u}}_{{}_{k}}^{n+1}(\omega )=\underset{{\hat{u}}_{k}(\omega )}{\arg\min}f(\omega )$. Let $\Delta (\omega )=\hat{x}(\omega )-\sum_{i\neq k}{\hat{u}}_{i}(\omega )+\hat{\lambda }(\omega )/2$, then:

(10) $$\left\{\begin{array}{l} \frac{\partial f(\omega )}{\partial {\hat{u}}_{k}(\omega )}=(4\alpha {\left(\omega -\omega_{k}\right)}^{2}+2)u_{k}^{*}(\omega )-2\Delta^{*}(\omega )=0\\ \frac{\partial f(\omega )}{\partial {\hat{u}}^{*}{}_{k}(\omega )}=(4\alpha {\left(\omega -\omega_{k}\right)}^{2}+2)u_{k}(\omega )-2\Delta (\omega )=0 \end{array}\right.$$

The minimum value of ${\hat{u}}_{{}_{k}}^{n+1}(\omega )$ can be modelled:

(11) $${\hat{u}}_{{}_{k}}^{n+1}(\omega )=\frac{\Delta (\omega )}{1+2\alpha {\left(\omega -\omega_{k}\right)}^{2}}=\frac{\hat{x}(\omega )-\sum_{i\neq k}{\hat{u}}_{i}(\omega )+\hat{\lambda }(\omega )/2}{1+2\alpha {\left(\omega -\omega_{k}\right)}^{2}}$$

where ${\hat{u}}_{{}_{k}}^{n+1}(\omega )$ is the Wiener filter of the current remainder $\hat{x}(\omega )-\sum_{i\neq k}{\hat{u}}_{i}(\omega )$, and $\omega_{k}$ represents the center of the mode power spectrum. In the same way, the minimum value of the central frequency can be obtained, that is, the least square linear regression frequency estimation of the center frequency in the mode can be obtained.

(12) $${\hat{\omega }}_{k}^{n+1}(\omega )=\frac{\int_{0}^{\infty }\omega {\left|{\hat{u}}_{k}(\omega )\right|}^{2}d\omega }{\int_{0}^{\infty }{\left|{\hat{u}}_{k}(\omega )\right|}^{2}d\omega }$$

To sum up, the frequency domain updating formulas and convergence conditions of ${\hat{\xi }}_{k}^{n+1}(\omega )$, ${\hat{u}}_{k}^{n+1}(\omega )$ and ${\hat{\lambda }}^{n+1}(\omega )$ are as follow:

(13) $$\left\{\begin{array}{l} {\hat{u}}_{k}^{n+1}\left(\omega \right)\leftarrow \frac{\hat{x}\left(\omega \right)-\sum_{i<k}{\hat{u}}_{i}^{n+1}\left(\omega \right)-\sum_{i>k}{\hat{u}}_{i}^{n}\left(\omega \right)+\frac{{\hat{\lambda }}^{n}\left(\omega \right)}{2}}{1+2\alpha {\left(\omega -\omega_{k}\right)}^{2}}\\ {\hat{\omega }}_{k}^{n+1}\left(\omega \right)\leftarrow \frac{\int_{0}^{\infty }\omega {\left|{\hat{u}}_{k}\left(\omega \right)\right|}^{2}d\omega }{\int_{0}^{\infty }{\left|{\hat{u}}_{k}\left(\omega \right)\right|}^{2}d\omega }\\ {\hat{\lambda }}^{n+1}\left(\omega \right)\leftarrow {\hat{\lambda }}^{n}\left(\omega \right)+\tau \left[\hat{x}\left(\omega \right)-\sum_{k}{\hat{u}}_{k}^{n+1}\left(\omega \right)\right]\\ \sum_{k}{\left\|u_{k}^{n+1}(\omega )-u_{k}^{n}(\omega )\right\|}_{2}^{2}/{\left\|u_{k}^{n}(\omega )\right\|}_{2}^{2}<\varepsilon \end{array}\right.$$

When the end of iteration condition is satisfied, the frequency band is adaptively segmented according to the frequency characteristics of the signal. Then, the inverse Fourier transform is performed on ${\hat{u}}_{k}(\omega )$, and the real part is the desired $u_{k}(t)$. The variational mode of a real signal can be obtained by VMD, and the Hilbert transform of BLIMF is performed to obtain the instantaneous frequency and amplitude, namely Hilbert spectrum.

## 3. Complex Variational Mode Decomposition

**Figure 1:**
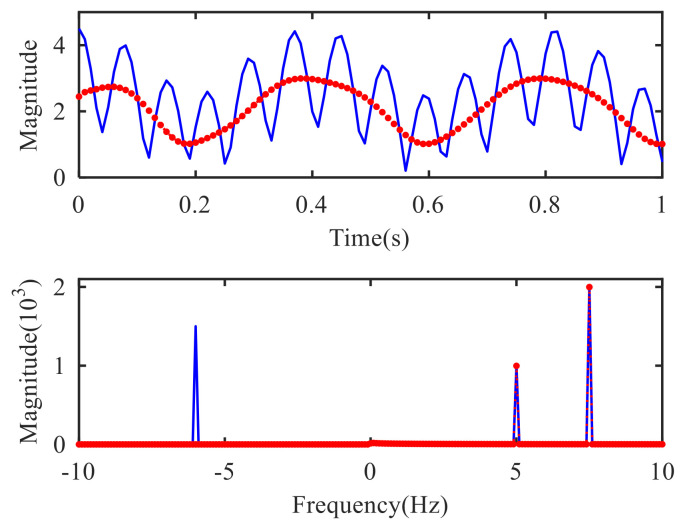
Original signal (blue solid line) and reconstruction signal by VMD (red dashed line).

The VMD mathematical theory demonstrates that the algorithm is only suitable for real signal analysis. After Hilbert transform, the two-side spectrum of a real signal will be transformed into a single-side spectrum. Due to the symmetry of the two-side spectrum of the real signal, the spectrum structure of the reconstructed signal will not be changed after being converted into a single-side spectrum and the amplitude will become twice as much as before. In radar signal processing, the echo is a complex signal, and the spectrum of that is single-side and asymmetric. If VMD were utilized to decompose the echo directly, half of the spectrum structure of the reconstructed signal would be lost, and only the partial signal corresponding to the positive frequency would be reconstructed. As the simple complex signal $S(t)=e^{j}{}^{10\pi t}+1.5e^{-j12\pi t}-2e^{j15\pi t}$, for example, the time domain and frequency domain results of the original signal and VMD reconstructed signal are shown in Figure 1. In the reconstructed signal spectrum by VMD, the positive frequency component is reconstructed accurately, but the frequency loss occurs at f=−6Hz, that is to say, the reconstruction of the negative frequency component fails.

In view of the defect that VMD cannot process complex signals, Reference [19] extended the VMD algorithm to the complex domain by decomposing the complex signal into real part and imaginary part. After VMD processing twice, the decomposed modes were linearly superimposed to reconstruct the original signal successfully. However, this CVMD method needs to construct a band-pass filter to separate the real part and the imaginary part of the complex signal. Not only is this method more complicated, but it also causes phase difference between the reconstructed signal and the original signal. In this paper, a novel CVMD method is proposed by analyzing the spectrum of complex signals. In frequency spectrum analysis, the major difference between the real signal and the complex signal is that the complex signal has negative frequency component. If the negative frequency component is converted into a real signal separately through frequency spectrum analysis, then the complex signal can be converted into two real signals for further processing. The proposed method can complete complex signal processing. The steps are as follows:

(1) The complex signal S(t) can be transformed into frequency domain by Fourier transform, which is recorded as S(ω). By setting the negative frequency axis or positive frequency axis of S(ω) to zero respectively, the positive frequency part $S_{+}(\omega )$ and negative frequency part $S_{-}(\omega )$ of S(ω) are taken out, and the signal length cannot be changed, written as:

(14) $$S(\omega )=S_{+}(\omega )+S_{-}(\omega )$$

At this time, $S_{+}(\omega )$ and $S_{-}(\omega )$ are a typical single-side spectrum. But for real signals, the negative frequency is meaningless. Therefore, it is necessary to preprocess $S_{-}(\omega )$. The simplest processing method is rearranging $S_{-}(\omega )$ in reverse order. After this step, the negative frequency component is mapped to the corresponding positive frequency, namely:

(15) $${S^{\prime }}_{-}(\omega )=S_{-}(-\omega )$$

This method can ensure that the spectrum structure does not change, and all the original negative frequencies are symmetrically placed on the positive frequency axis.

(2) After inverse Fourier transform of $S_{+}(\omega )$ and ${S^{\prime }}_{-}(\omega )$, the corresponding time domain signals $S_{+}(t)$ and ${S^{\prime }}_{-}(t)$ can be obtained to decompose, this is because both $S_{+}(t)$ and ${S^{\prime }}_{-}(t)$ are real signals at this moment. Besides, since the signal length is not changed during spectrum processing, the corresponding time domain signals $S_{+}(t)$ and ${S^{\prime }}_{-}(t)$ have the same length as the original signal S(t).
(3) VMD is used to decompose $S_{+}(t)$ and ${S^{\prime }}_{-}(t)$ to get the corresponding BLIMF components ${\hat{u}}_{+}(\omega )$ and ${{\hat{u}}^{\prime }}_{-}(\omega )$. Since ${\hat{u}}_{+}(\omega )$ and ${{\hat{u}}^{\prime }}_{-}(\omega )$ only contain positive frequency, if the inverse Fourier transform were applied to BLIMF component directly, the reconstructed signal would still contain positive frequency, which is inconsistent with the real situation. This is caused by making ${S^{\prime }}_{-}(\omega )=S_{-}(-\omega )$. Therefore, in order to ensure that the reconstructed signal is consistent with the original signal, it is necessary to carry out reverse order rearrangement to signal ${{\hat{u}}^{\prime }}_{-}(\omega )$, namely:

(16) $${\hat{u}}_{-}(\omega )={{\hat{u}}^{\prime }}_{-}(-\omega )$$

Finally, the inverse Fourier transform is implemented on ${\hat{u}}_{+}(\omega )$ and ${\hat{u}}_{-}(\omega )$ to obtain $u_{+}(t)$ containing only positive frequency and $u_{-}(t)$ containing only negative frequency. Thus, the constructed signal can be written as:

(17) $$S(t)=u_{+}(t)+u_{-}(t)$$

**Figure 2:**
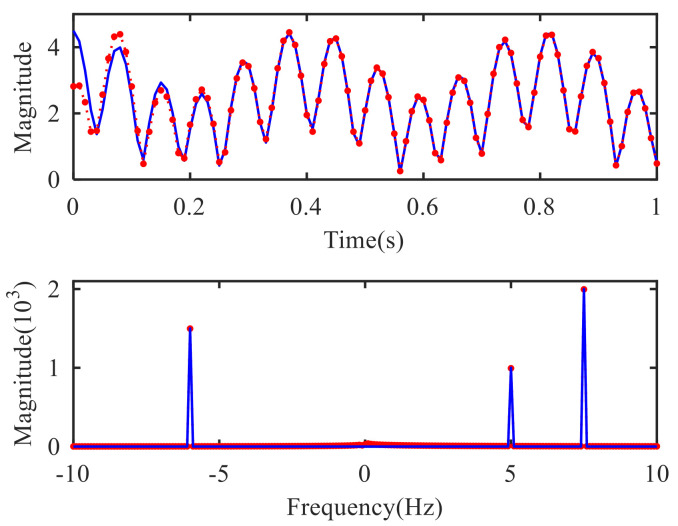
Original signal (blue solid line) and reconstruction signal by CVMD (red dashed line).

As seen in Figure 2, both positive frequency components and negative frequency components are reconstructed correctly. Therefore, the CVMD method proposed in this paper can extend the VMD method to the complex domain and process the complex signal without interference on the phase of the original signal. Due to the fact that the CVMD algorithm is based on VMD algorithm, there are no drawbacks such as modal aliasing or end-point effect. But since the VMD algorithm is used twice in this processing, the calculation amount of the CVMD algorithm is about twice as much as the VMD algorithm.

## 4. Adaptive Complex Variational Mode Decomposition

### 4.1. Solution of Optimal Decomposition Layer K

In recent years, the EMD method has been widely used in the field of signal processing. Due to the utilization of recursive, this method can adaptively decompose the signal, and the number of the decomposed modes is only affected by the signal itself. Unlike the EMD method, both VMD and CVMD need to set the decomposition layer *K* in advance. If the value of *K* is set too small, it will lead to under-decomposition. On the contrary, if it is too large, it will lead to over-decomposition. This is one of the main limitations of the VMD algorithm. Currently, most selection methods of *K* are based on experience [20,21]. Thus, the focus is how to determine the best *K* value. By using the method of detrended fluctuation analysis (DFA) to calculate the long-range correlation scaling index $\alpha_{0}$ of time series [22], the threshold of scaling index was set to judge the number of scaling indexes of modal components which were larger than the threshold value, and then the mode number can be ensured by the correlation model between decomposition layer *K* and scaling index $\alpha_{0}$. The core of this method is DFA, but this method has some limitations in dealing with the fluctuation trend of signal, thus it is less robust. In Reference [23], a feedback-based variational mode decomposition is proposed, in which the initial value *K* = 2 is set for the two-mode decomposition, and the similarity coefficient is used to measure the purity of the mode, then the purest mode is fed back into the input of the signal and subtracted from the original signal. Finally, the iterative termination condition is used to determine whether the decomposition continues or not. This method can avoid the problem of setting the value of *K*, but it uses VMD several times in iteration, therefore, the computation is complicated. Furthermore, the performance of mode separation is worse for those with similar fundamental frequencies. Reference [24] considered that the modes decomposed by VMD are orthogonal, so the linear sum of each component’s energy is equal to the energy of the original signal. When under-decomposition or over-decomposition occurs, the linear sum of the mode component’s energy is less than or greater than the energy of the original signal. Therefore, the optimal decomposition layer can be determined by comparing the linear sum of the mode energy with the energy of the original signal. If the two values are equal, the *K* would be ensured, but the mode decomposed by VMD is not always completely orthogonal, and there is a large error in this method. In Reference [25], the sample entropy of each mode is used to judge whether the over-decomposition occurs or not, and the correlation is used to judge whether to retain the over-decomposition modes or not, which weakens the over-decomposition to a certain extent. However, the under-decomposition is not discussed. Reference [26] considered obtaining the *K* value of VMD by using the number of modes after EMD, but the number of modes is not accurate when the mode aliasing occurs.

According to the principle of SVD, the size of a singular value of signal directly reflects the composition of the signal. After signal processing by SVD, the several larger singular values reflect the principal components of the signal usually, including the clutter and the target, and then the smaller singular values reflect the noise component. While the CVMD algorithm divides the signal into *K* components according to the rules, the residual component is the interference noise. It can be seen that SVD and CVMD have similar functions in signal processing, so the optimal decomposition layer of CVMD can be determined by the order of effective singular values. Then, the problem of finding the optimal decomposition layer of CVMD can be transformed into the order of effective singular values searching. The steps are as follows:

(1) Constructing the Hank matrix H [27] by using the signal S(t).

The signal sequence $S=[s_{1},s_{2},\ldots ,s_{M}]$ with length M is embedded into the reconstruction m vectors $S_{i}=[s_{(i-1)\kappa +}{}_{1},s_{(i-1)\kappa +}{}_{2},\ldots ,s_{L+}{{}_{(i-1)\kappa +}}_{1}],i=1,2,\ldots ,m$ according to a certain delay κ, where L=M−(i−1)κ−1. Then, the Hank matrix H with L×m dimension is constructed as follows:

(18) $$H=\left[\begin{array}{c} S_{1}\\ S_{2}\\ \vdots \\ S_{m} \end{array}\right]=\left[\begin{array}{cccc} s_{1} & s_{2} & \cdots & s_{L+1}\\ s_{\kappa +1} & s_{\kappa +2} & \cdots & s_{L+\kappa +1}\\ \vdots & \vdots & & \vdots \\ s_{(m-1)\kappa +}{}_{1} & s_{(m-1)\kappa +}{}_{2} & \cdots & s_{M} \end{array}\right]$$

(2) Implement the SVD processing on H to obtain the singular values and the slope of the singular value.

In the construction of Hank matrix H, it usually satisfies m<L. Therefore, Hank matrix H can be expressed as:

(19) $$H=U\lambda V^{H}$$

where λ is a singular value matrix with L×m dimension, its main diagonal element is $\lambda_{i}$ and other elements are equal to zero, namely $\lambda =diag[\lambda_{1},\lambda_{2},\ldots ,\lambda_{m}]$ and $\lambda_{1}\geq \lambda_{2}\geq \ldots \geq \lambda_{m}\geq 0$. The matrix U is eigen-column vectors of λ, and matrix $V^{H}$ is eigen-row vectors of λ.

According to the curve of singular value distribution, the slope $g_{m}$ of the corresponding singular value is calculated by:

(20) $$g_{m}=\frac{d\lambda }{dm}$$

(3) Setting the amplitude ratio threshold to search the steady-state starting position of the slope, that is the effective singular value order.

At present, many scholars still rely on experience to find the effective singular value order. This is for the reason that the mutation point of the actual signal singular value curve is not easy to be realized by the algorithm, and it is sensitive to noise. Thus, it is not rigorous to set the threshold to find the effective singular value order directly. In other words, the order of the effective singular value is the position where the trend of the singular value curve changes. The problem can be transformed into searching the starting position where the slope tends to be stable.

Firstly, finding the maximum $g_{m-\max}$ and minimum $g_{m-\min}$ of slope $g_{m}$ and defining the amplitude ratio function z and the amplitude ratio threshold $\gamma_{1}$:

(21) $$z=\frac{g_{m-\min}}{g_{m-\max}}$$

When $z<\gamma_{1}$, the slope $g_{m}$ fluctuates greatly, and $g_{m-\max}$ will be set to zero. Then, a new slope ${g^{\prime }}_{m}$ is obtained, and new amplitude maximum of ${g^{\prime }}_{m-\max}$ and amplitude ratio $z^{\prime }=g_{m-\min}/{g^{\prime }}_{m-\max}$ will be generated again. Until the slope ${g^{\prime }}_{m}$ amplitude ratio function satisfies $z^{\prime }\geq \gamma_{1}$, the iteration stops. It is considered that the new slope ${g^{\prime }}_{m}$ tends to be stable, and the position of the minimum non-zero point in the slope ${g^{\prime }}_{m}$ is the searched order of the effective singular value.

### 4.2. Principle of Signal Reconstruction

Another serious problem faced by time-frequency analysis methods is the selection of the signal reconstruction mode. At present, there are mainly the cross-correlation (CORR) judgment method [28], information entropy judgment method [29], energy proportional judgment method [30], and probability density function judgment method [31]. Among these, the cross-correlation judgment method and probability density function judgment method have better robustness. The judgment method of the probability density function is discussed in Reference [32]. Firstly, the probability density functions of the original signal and each mode are estimated. Then, the Euclidean distance (ED), Bhattacharyya distance (BD), and Hausdorff distance (HD) between the probability density functions of the original signal and each mode are calculated respectively to measure the correlation. Finally, it gives the conclusion that the Hausdorff distance has the optimal effect on measuring the correlation of the two probability density functions. However, in the field of radar signal processing, in addition to the effective target signal, there are also noise and clutter in the signal. In this case, Hausdorff distance can easily cause mismatching.

In order to select the correlated modes for reconstruction after CVMD decomposition efficiently, this paper uses Mahalanobis distance [33] to measure the correlation between each mode and the original signal. Mahalanobis distance can fully consider the correlation of the size and characteristics between two vectors. The Mahalanobis distance does not distinguish data types and does not need to estimate the probability density function. The calculation formula is as follows:

(22) $$D=\sqrt{{\left(X-\mu \right)}^{T}\Sigma^{-1}(X-\mu )}$$

where D represents the Mahalanobis distance between the sample to be tested and the reference sample, X is the sample to be tested, μ is the mean value of the reference sample, and Σ is the covariance estimation of the reference sample. The larger D, the weaker correlation between the two samples, and the smaller D, the stronger correlation between the two samples.

The Mahalanobis distance $D_{i}$ of each mode is calculated and compared with its mean value $\bar{D}=\frac{1}{K}\sum_{i=1}^{K}D_{i}$ to preliminarily judge the correlation between each mode and the original signal.

(23) $$\left\{\begin{array}{l} P_{n}=BLIMF_{i}D_{i}<\bar{D}\\ Q_{n}=BLIMF_{i}D_{i}\geq \bar{D} \end{array}\right.$$

In the Equation (23), $P_{n}$ can be judged as strong correlation mode and $Q_{n}$ as weak correlation mode. The second judge is designed to avoid the conditions where all the points in the Mahalanobis distance curve are very close or the last point larger than mean value on the curve is pretty close to the mean value, which will generate the error judgment of classifying the strong correlation mode to the weak correlation mode.

This idea set the threshold value $\gamma_{2}$ to rejudge the mode Q, which is larger than $\bar{D}$ and closest to $\bar{D}$, if it satisfies:

(24) $$\frac{\bar{D}}{Q_{i}}>\gamma_{2}$$

The mode Q is reclassified to the strong correlation mode, otherwise the original judgment is kept unchanged. At this time, the radar echo separation is realized by selecting the strong correlation mode $P_{n}$ and $Q_{i}$ for local reconstruction, written as follows:

(25) $$S(t)=\sum_{n=1}^{N}P_{n}+Q$$

**Figure 3:**
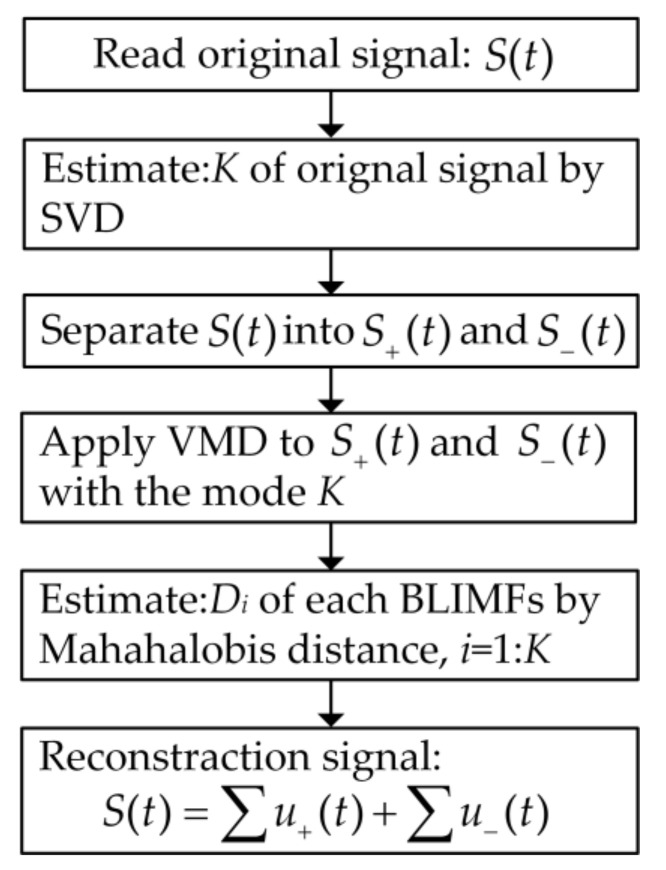
The flowchart of the adaptive CVMD algorithm.

In summary, the flowchart of the proposed adaptive CVMD algorithm is as shown in Figure 3.

## 5. Results and Discussions 

### 5.1. Analysis of Experimental Data

**Figure 4:**
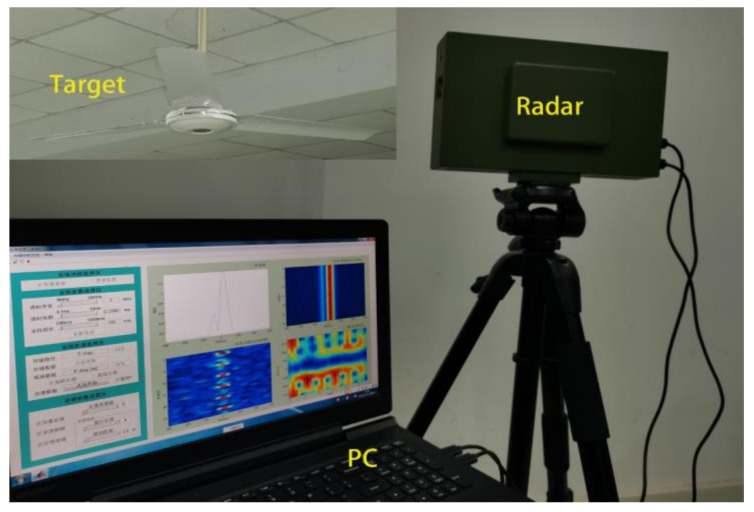
Experimental platform.

In order to verify the effectiveness of the CVMD algorithm in the field of radar signal processing, an experimental platform was built for data acquisition, as shown in Figure 4. The experimental platform is mainly composed of radar mainframe and PC, and its target is a rotating fan driven by a motor. The parameters of radar are as follow: carrier frequency *f*c = 24 GHz, repetition frequency PRF = 4000 Hz, bandwidth B = 10 MHz and sampling frequency *f*s = 500 KHz. The parameters of the fan are as follow: the number of blades N = 3, the length of blades L = 0.6 m, the width of blades W = 0.12 m, the rated rotational speed ω = 320 rpm. The observation time of this experiment is 0.5 s, and the observation angle is $40^{\circ }$.

**Figure 5:**
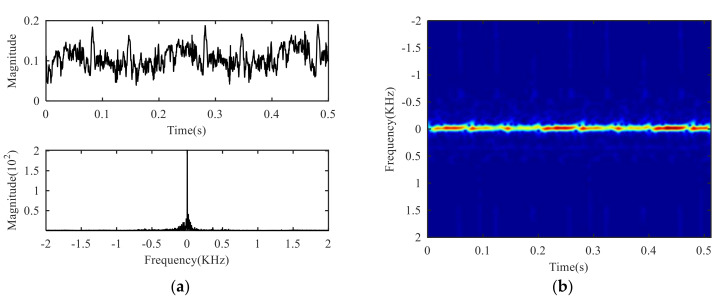
Experimental signal: (**a**) Time domain and frequency domain, (**b**) time-frequency domain.

The time domain waveform, frequency spectrum and time-frequency diagram of the signal collected by the experimental platform are shown in Figure 5. The flicker modulation caused by target rotation can hardly be observed in the time domain waveform, and only a strong peak near-zero frequency can be observed in the frequency spectrum. The law observed in the time-frequency diagram is consistent with that in the frequency domain. Therefore, it can be seen that the target echo is completely submerged in the clutter, so it is necessary to suppress the clutter.

**Figure 6:**
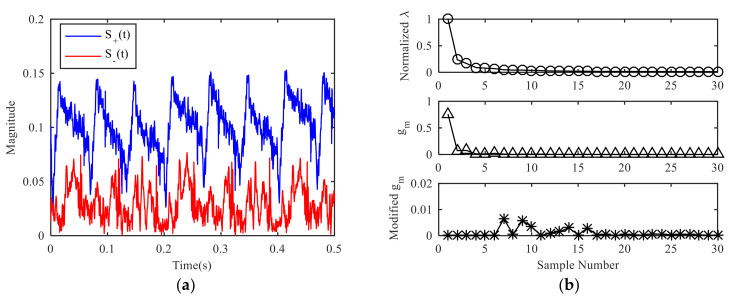
Solution of decomposition layer: (**a**) Newly constructed signal $S_{+}(t)$ and $S_{-}(t)$, (**b**) curve of effective singular value.

The preprocessing of the signal obtains two signals to be decomposed, as shown in Figure 6a. The obvious periodicity can be observed for both signals, and the signal $S_{+}(t)$ containing only positive frequency component is stronger than the signal $S_{-}(t)$ containing only negative frequency component. After the analysis of the radar signal, the positive frequency usually means that the target is moving close to the radar, while the negative frequency is moving away from the radar. When targets move close to the radar, the distance between the target and the radar becomes closer and the echo intensity is relatively strong. When targets move away from the radar, the distance between the target and the radar becomes longer and the echo intensity is relatively weak.

The SVD processing of $S_{+}(t)$ or $S_{-}(t)$ can obtain the singular value curve, and the slope curve of the singular value, as shown in Figure 6b. From the slope curve, it can be seen that the slope tends to be flat after several large values. It can be judged that the effective order of singular value is about 5–8. At this moment, the slope of singular value changes slowly, the specific order cannot be determined directly. The amplitude ratio function value is calculated iteratively, and the amplitude ratio threshold is set at $\gamma_{1}=0.01$, and if the point with large fluctuation is set to zero, the amplitude of the singular value slope curve will be limited to a small range. At this time, the effective order of the singular value is determined as 7 by finding the first non-zero position.

In order to discuss the performance of the SVD-based method under different SNR conditions, noise is added in this experiment. Under different SNR conditions ($\gamma_{1}$ is the optimal threshold), 100 of Monte Carlo simulation experiments are used to compare the *K* estimation effect of DFA, EMD and SVD methods. The concrete results are shown in Table 1. The table demonstrates that SVD has the best estimation effect, and this method is robust under lower SNR conditions.

**Figure 7:**
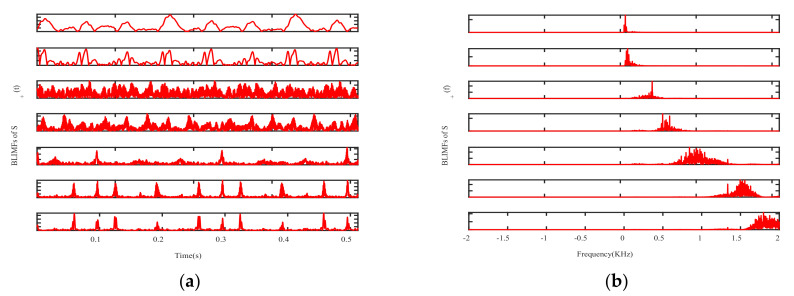

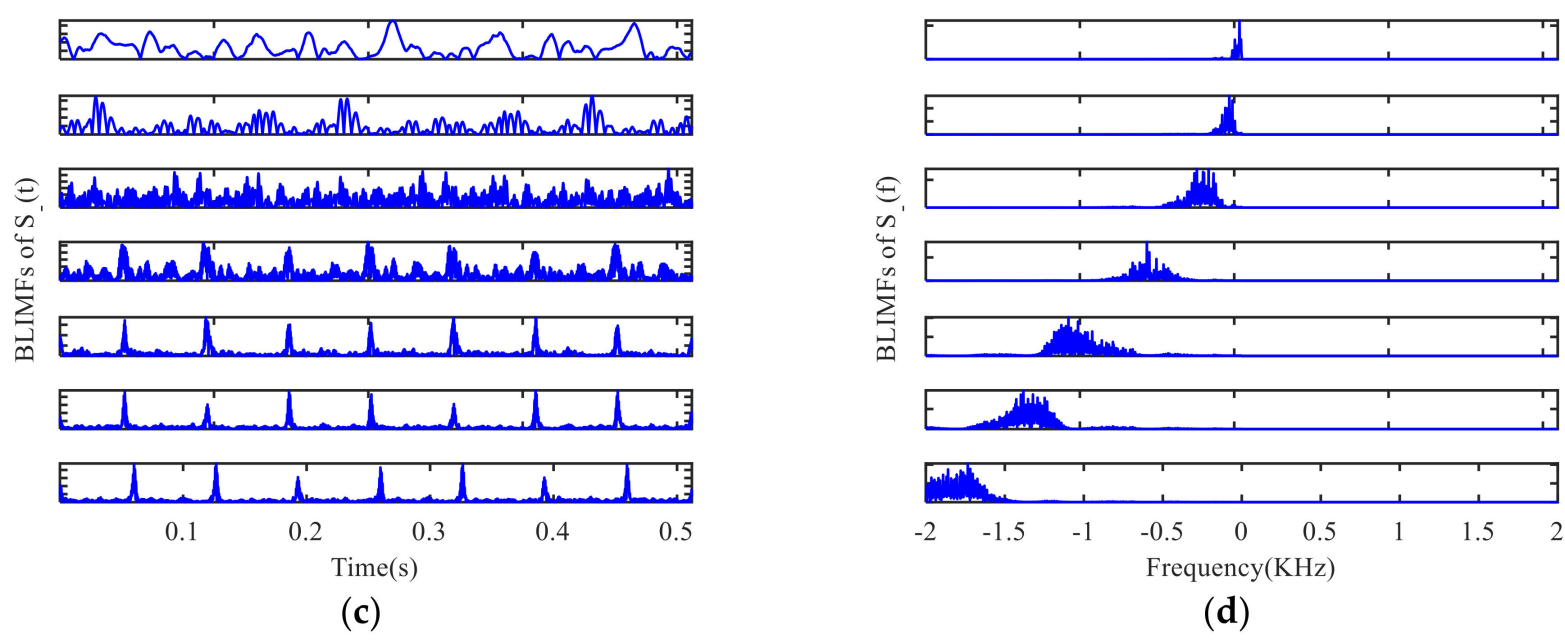
VMD of $S_{+}(t)$ and $S_{-}(t)$: (**a**) BLIMFs of $S_{+}(t)$ in time domain, (**b**) BLIMFs of $S_{+}(t)$ in frequency domain, (**c**) BLIMFs of $S_{-}(t)$ in time domain, (**d**) BLIMFs of $S_{-}(t)$ in frequency domain.

**Figure 8:**
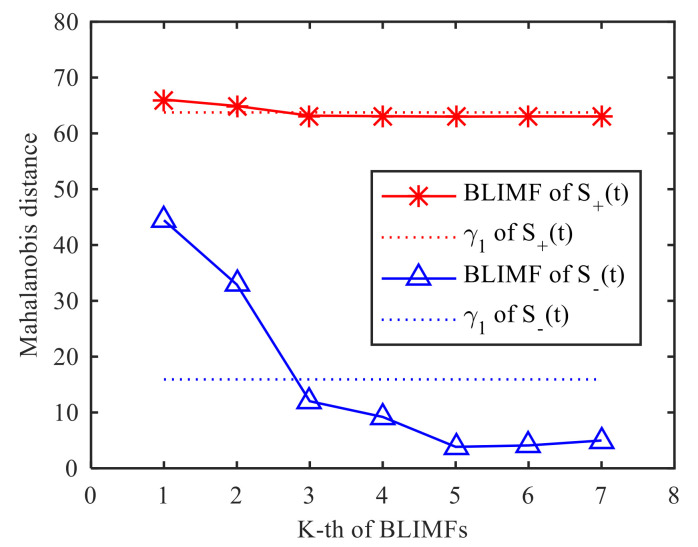
Mahalanobis distance between S(t) and the BLIMFs.

The effective order of singular value is used as the decomposition layer number of CVMD, that is K=7, after decomposition of the signals $S_{+}(t)$ and $S_{-}(t)$, and the modal components and their corresponding spectrum are shown in Figure 7. It can be seen from the spectrum that the signals decomposed by CVMD have a certain bandwidth and different central frequencies, and after decomposition, whether the modal components are obtained by $S_{+}(t)$ decomposition or the modal components obtained by $S_{-}(t)$ decomposition, there is no under-decomposition or over-decomposition in the spectrum, so we can judge that the K is the best value, which shows that the effective order of singular values of SVD is equivalent to the optimal decomposition layer of CVMD. Figure 8 is the Mahalanobis distance curve of each modal component to the original signal. By comparing with the mean of Mahalanobis distance, the strongly correlated mode and the weakly correlated mode can be roughly identified. By setting a threshold value of $\gamma_{2}=0.99$ to determine whether a false judgment occurs, in fact, it is only when all the points on the Mahalanobis distance curve are close to each other, or when the last point on the curve is extremely close to the mean value, that a false positive can occur, it can be understood as a fault-tolerant threshold with limited impact on signal reconstruction performance. Table 2 gives the exact values of the cross-correlation coefficient, the Bhattacharyya distance, the Hausdorff distance, the Euclidean distance and the Mahalanobis distance. It can be seen from the comparison that the strongly correlated modes determined by calculating the cross-correlation coefficient are BLIMF4-BLIMF7 and BLIMF3-BLIMF4 respectively, by calculating the Bhattacharyya distance, the strong correlation modes determined by Hausdorff distance and Euclidean distance are BLIMF5-BLIMF7, and those determined by Mahalanobis distance are BLIMF3-BLIMF7, and it was found that only the Mahalanobis distance can accurately separate the micro-motion signals.

**Figure 9:**
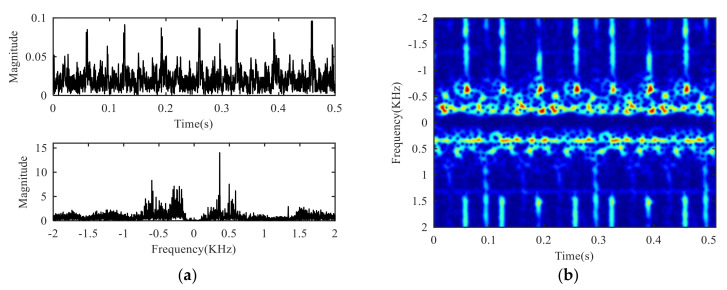
Reconstruction signal: (**a**) Time domain and frequency domain, (**b**) time-frequency domain.

The time domain waveform, frequency spectrum and time-frequency diagram of the reconstructed signal are shown in Figure 9. At this time, periodic peaks can be seen on the time domain waveform, that is the flicker characteristics caused by the target micro-motion [34]. It can be seen from the spectrum and time-frequency diagram that the signal near the zero frequency is suppressed, that is, the clutter is suppressed successfully, and the separation of the icro-motion signal and the clutter is realized. Due to the target position being fixed in the experiment, the fuselage signal is still at zero frequency, thus, the separation of the fuselage signal and the icro-motion signal has been accomplished.

### 5.2. Analysis of Measured Data

**Figure 10:**
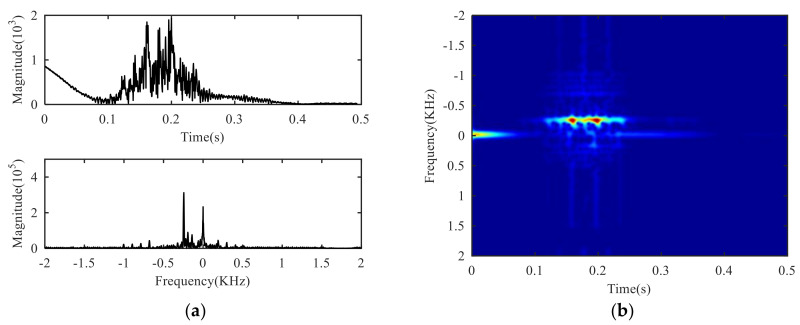

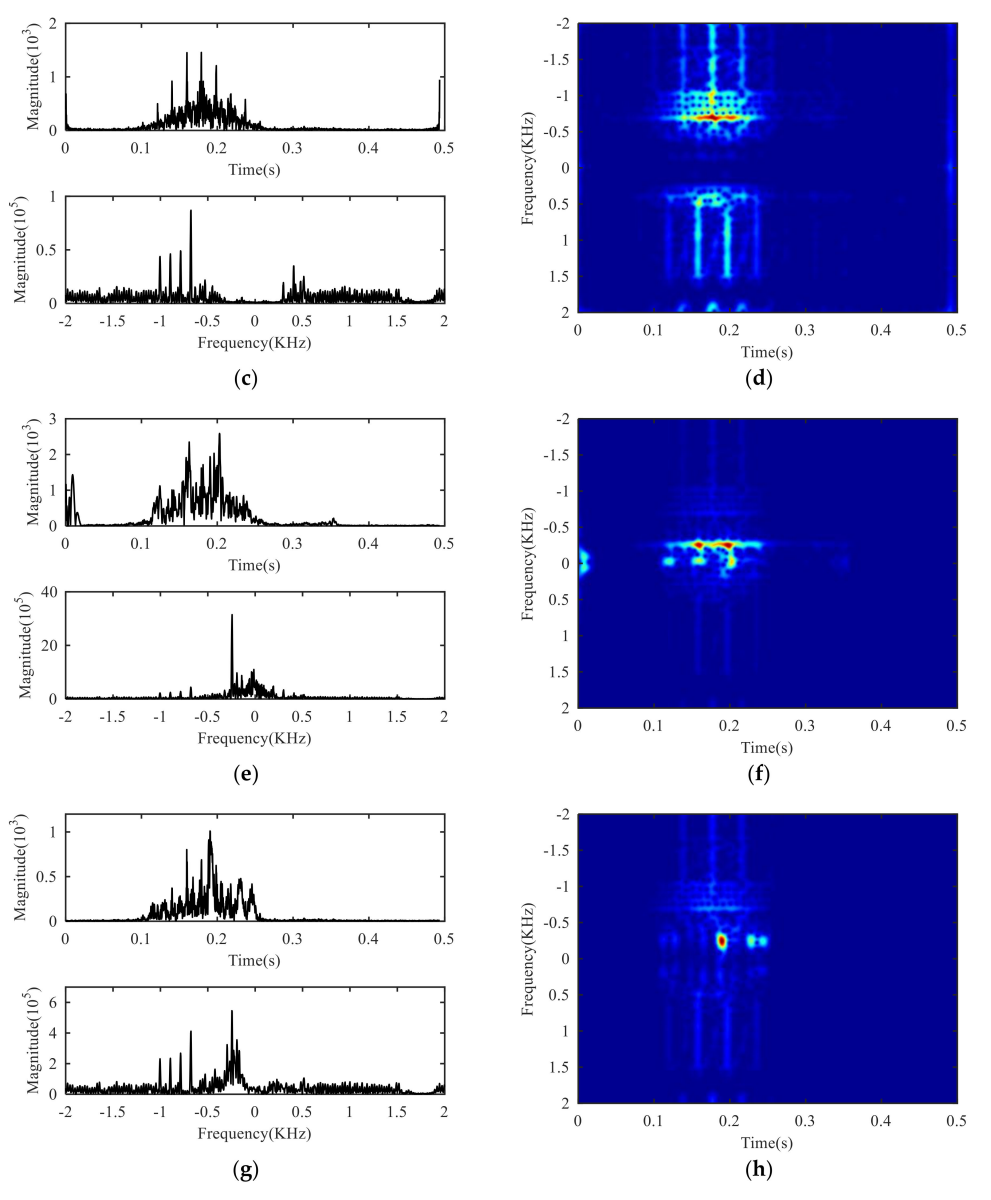

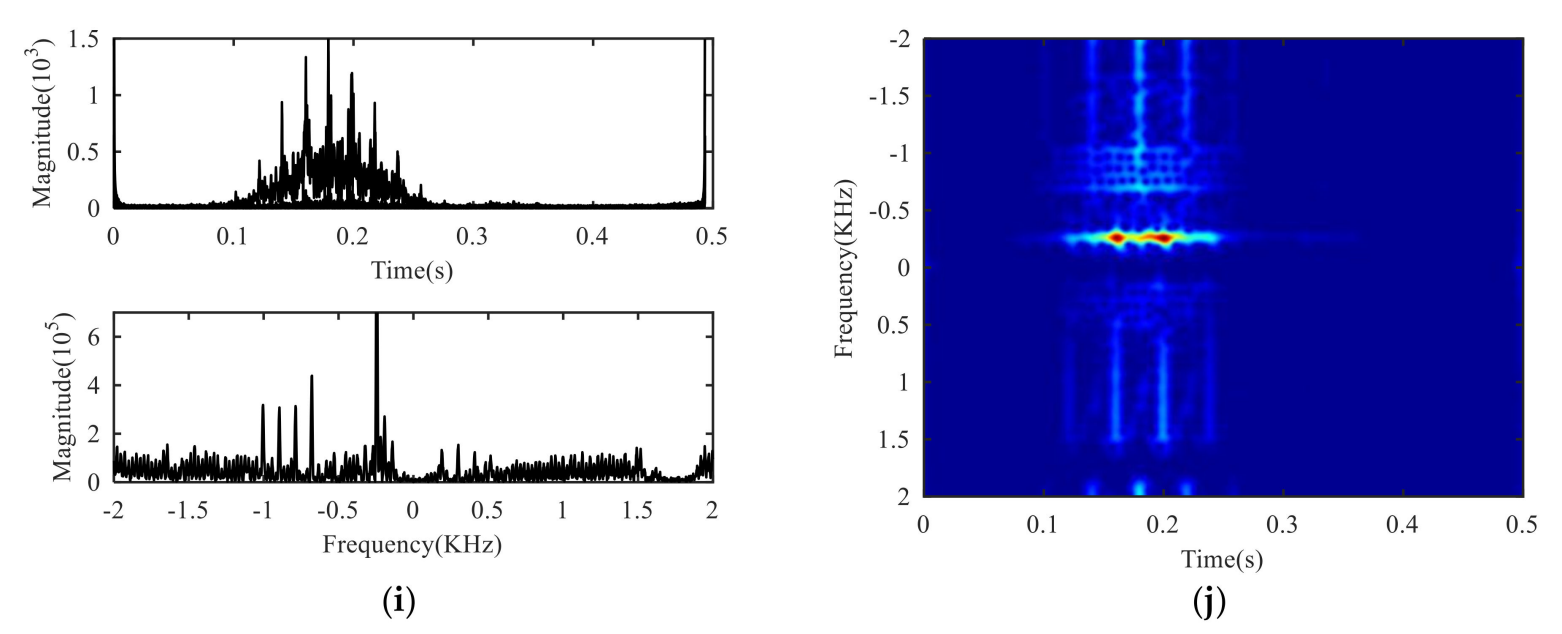
Analysis of helicopter data: (**a**) Original signal in time domain and frequency domain, (**b**) original signal in time-frequency domain, (**c**) reconstruction in time domain and frequency domain by CVMD, (**d**) reconstruction in time-frequency domain by CVMD, (**e**) reconstruction in time domain and frequency domain by LMD, (**f**) reconstruction in time-frequency domain by LMD, (**g**) reconstruction in time domain and frequency domain by EMD, (**h**) reconstruction in time-frequency domain by EMD, (**i**) result in time domain and frequency domain by MTI, (**j**) result in time-frequency domain by MTI.

**Figure 11:**
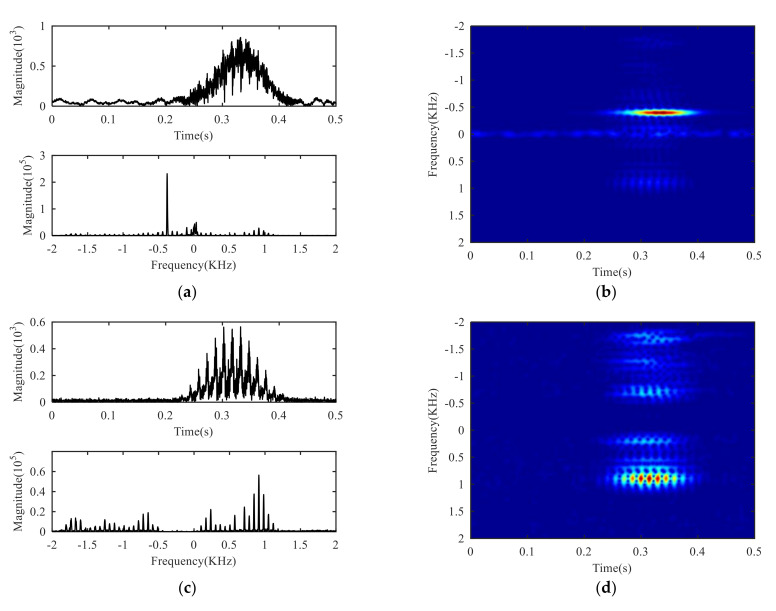

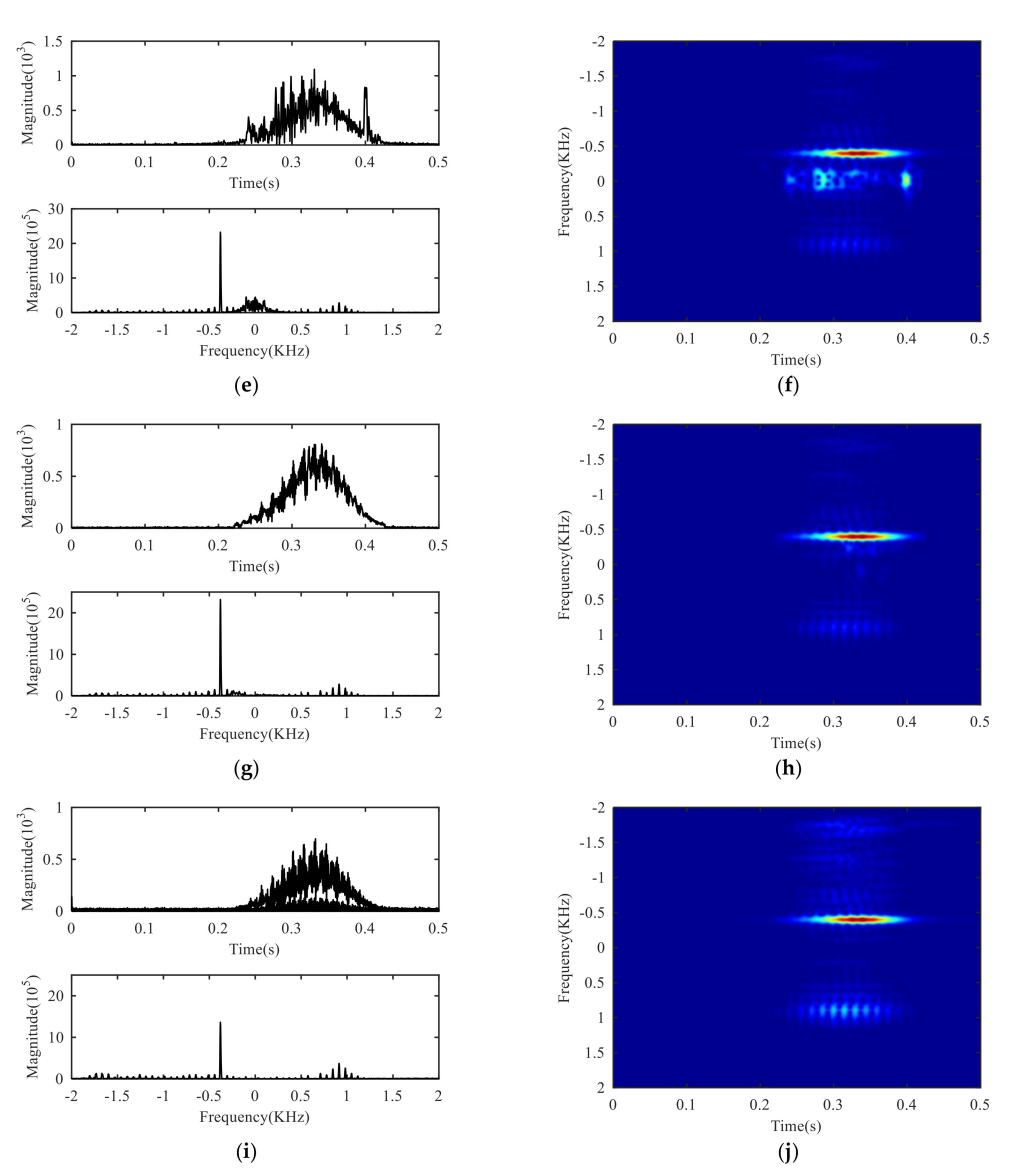
Analysis of propeller data: (**a**) Original signal in time domain and frequency domain, (**b**) original signal in time-frequency domain, (**c**) reconstruction in time domain and frequency domain, (**d**) reconstruction in time-frequency domain, (**e**) reconstruction in time domain and frequency domain by LMD, (**f**) reconstruction in time-frequency domain by LMD, (**g**) reconstruction in time domain and frequency domain by EMD, (**h**) reconstruction in time-frequency domain by EMD, (**i**) result in time domain and frequency domain by MTI, (**j**) result in time-frequency domain by MTI.

In this part, two sets of measured data are directly selected and analyzed. The target in data 1 is a helicopter, and the target in data 2 is a transport aircraft. The time domain waveform, frequency spectrum and time-frequency diagram of the original signal of data 1 and the result of adaptive CVMD processing are shown in Figure 10a–d. Figure 10e–j is the time domain waveform, frequency spectrum and time frequency diagram of LMD processing, EMD processing and MTI processing. Figure 11a–d is the result of adaptive CVMD processing of data 2, and Figure 11e–j is the result of LMD processing, EMD processing and MTI processing of data 2.

From the processing results of two sets of radar measured data of different micro-motion targets, it can be seen that the adaptive CVMD method can effectively separate the clutter and the fuselage component of the target in the echo and reserve its fretting component. In Figure 10c and Figure 11c, the flicker of the reconstructed signal in the time domain is obvious, a notch appears on the frequency spectrum at the zero frequency and the doppler position of the fuselage, which means the echo is successfully separated. It is shown that the number of decomposition modes and the selection of reconstructed modes of CVMD are reasonable and practical. Due to the presence of high-energy clutter and fuselage on the original time-frequency diagram of Figure 10b and Figure 11b, the micro-motion characteristics of the target can hardly be observed, in Figure 10d and Figure 11d, the micro-motion characteristics of the target are clearly visible, which provides favorable conditions for further research on micro-motion feature extraction and target recognition.

Comparing the results in Figure 10e–h and Figure 11e–h, the LMD and EMD methods can only suppress the clutter to a certain extent when dealing with the separation of the measured data. There is a certain residual clutter, and the separation effect of the fuselage is poor. Comparing the results in Figure 10i,j and Figure 11i,j, the MTI methods can totally suppress the clutter, but cannot separate the fuselage of the target, and this method has nonlinear amplification effect on high-frequency components affected by amplitude frequency characteristics. The result shows that the adaptive CVMD method presented in this paper is more robust and more practical than LMD, EMD and MTI methods in narrowband radar target micro-motion echo separation.

## 6. Conclusions

In order to solve the problem of VMD algorithm’s inability to process complex signal, the number of decomposition layer needs to be set manually and the signal reconstruction mode selection is difficult, therefore, an adaptive complex variational mode decomposition algorithm was proposed. Firstly, the complex signal was preprocessed in the frequency domain, and two new signals containing only positive frequency components and negative frequency components were obtained. Then, singular value decomposition was performed on the decomposed signal. The effective singular value order was found by setting the amplitude ratio threshold of the singular value slope as the optimal decomposition layer of CVMD. The optimal decomposition layer was used to decompose the two decomposed signals to obtain their respective modal components. The correlation of each mode was judged by calculating the Mahalanobis distance between each mode and the decomposed signal. Finally, the effectiveness of the method was verified by the experimental platform, and the robustness and practicability of the method were verified by comparing two groups of radar measured data with LMD, EMD and MTI methods. Through the above work, conclusions can be obtained as follows:

(1) The positive spectrum and negative spectrum were obtained by dividing the spectrum of the complex signal. The negative spectrum after reverse processing and positive spectrum were converted into time domain and sent to VMD for processing. All the decomposed modes were linearly superimposed to restore the original complex signal and realize CVMD processing of complex signal, but the computational complexity was doubled.
(2) The SVD was used to get the singular value vector of the decomposed signal. By setting the amplitude ratio threshold on the singular value slope curve, the effective singular value order can be obtained as the decomposition layer of CVMD. Under this decomposition layer, the CVMD decomposition result did not appear as under-decomposition or over-decomposition phenomenon, which shows that the effective singular value order of SVD is consistent with the optimal decomposition layer of CVMD.
(3) Mahalanobis distance can robustly judge the correlation between each mode and the original signal and can effectively highlight the strong correlation mode. By selecting the strong correlation mode to reconstruct the signal, it can achieve the separation of the micro-motion signal.

## Figures and Tables

**Table 1:** Comparison of the SVD with DFA and EMD for solving *K*.

SNR (dB)	−10	−8	−6	−4	−2	0	2	4	6	8	10
DFA	12	10	9	9	7	5	5	5	4	4	4
EMD	7	6	7	7	6	6	7	6	6	6	6
SVD	1	1	2	3	3	4	4	4	4	4	4

**Table 2:** Comparisons of different algorithms.

	CORR		BD		HD		ED		MD	
	$S_{+}(t)$	$S_{-}(t)$	$S_{+}(t)$	$S_{-}(t)$	$S_{+}(t)$	$S_{-}(t)$	$S_{+}(t)$	$S_{-}(t)$	$S_{+}(t)$	$S_{-}(t)$
BLIMF1	94.84	121.48	8.19	9.52	28.64	23.14	115.05	51.54	66.07	44.44
BLIMF2	114.43	132.73	8.21	9.37	45.67	40.71	170.27	122.49	64.90	32.90
BLIMF3	201.16	225.28	10.00	10.69	86.80	77.82	462.68	294.08	63.17	12.01
BLIMF4	234.21	248.68	9.68	10.49	158.36	146.12	670.03	477.20	63.08	9.20
BLIMF5	291.46	332.49	8.16	9.37	475.27	684.46	1290.7	1431.8	63.00	3.84
BLIMF6	244.87	296.81	8.02	8.68	468.58	622.35	1095.9	1266.6	63.04	4.10
BLIMF7	251.63	273.10	7.88	8.67	591.37	666.56	1231.1	1135.1	63.03	4.98

## Data Availability

Data sharing not applicable.
